# Pregnancy, childbirth, and birth in the state of Rio de Janeiro

**DOI:** 10.11606/s1518-8787.2025059supl1ap

**Published:** 2025-10-20

**Authors:** Maria do Carmo Leal

**Affiliations:** I Fundação Oswaldo Cruz. Escola Nacional de Saúde Pública. Departamento de Métodos Quantitativos em Saúde. Rio de Janeiro, RJ, Brasil

The World Health Organization's theme for World Health Day, observed on April 7, 2025, was "Healthy Beginnings, Hopeful Futures." This initiative called on governments and the global health community to prioritize the reduction of preventable maternal and neonatal deaths^1^. Beyond mortality reduction, the theme emphasized a broader concept of health, focusing on promoting the well-being of women and their infants to ensure a hopeful and prosperous future for both.

A sharp decline in fertility rates has been observed among Brazilian women, associated with increased educational attainment and greater access to the labor market^2,3^. Over the past decade, there has also been a notable reduction in adolescent pregnancies and a trend toward delayed motherhood among women aged 20 to 49^4^. This ongoing obstetric transition in the country^5^ suggests that, with fewer children and improved access to education and employment, Brazilian women are more likely to experience healthier pregnancies and give birth to healthier children.

Another critical factor influencing pregnancy outcomes is adequate access to high-quality prenatal and childbirth care that upholds women's sexual and reproductive rights. In Brazil, the Unified Health System (*Sistema Único de Saúde* – SUS) provides at least one prenatal visit to 99% of pregnant women. However, when considering the recommended number of visits relative to gestational age at birth, this percentage drops significantly^6^. Inadequate or absent prenatal care is associated with numerous adverse outcomes for both postpartum women and newborns^6^. Childbirth care, currently provided predominantly in hospital settings, has a coverage rate of 98%, although regional disparities persist, particularly in the North.

The overall scenario suggests that most women in Brazil are experiencing improved living conditions and receiving care during pregnancy. However, maternal and perinatal mortality rates have shown only minimal decline over the past two decades^7^. This underscores the need for a comprehensive analysis of pregnancy, childbirth, and birth across different national contexts. Given the profound social inequities present in the country, it is essential to examine whether these disparities are also reflected and perpetuated in the quality of care delivered within health services.

Policies implemented by the Ministry of Health to improve childbirth care in public maternity hospitals, such as the Humanization of Childbirth and Birth Program and the Stork Network (*Programa de Humanização do Parto e Nascimento e a Rede Cegonha*)^8,9^, have contributed to expanded access to appropriate obstetric technologies, enhanced comfort, and more dignified treatment for pregnant women and newborns^9^. However, national studies indicate that these advancements have not translated into a reduction in operative deliveries, which continue to increase, nor have they significantly impacted mortality rates^7,10^. Similarly, initiatives within the supplementary health sector, such as the Adequate Childbirth Program (Programa Parto Adequado), have shown promising results, although they remain in early stages of implementation^11^.

The thematic issue "Pregnancy, Childbirth, and Birth in the State of Rio de Janeiro" is part of the National Survey on Abortion, Childbirth, and Birth, known as Nascer no Brasil II (NB II). Funded by the Ministry of Health and the Oswaldo Cruz Foundation, the study includes interviews with 21,400 women in maternity hospitals. The sample design ensures representation across Brazil's major geographic regions (North, Northeast, Southeast, South, and Central-West), types of childbirth funding (public, private, and mixed), and areas of residence (metropolitan and non-metropolitan regions).

In the state of Rio de Janeiro (RJ), additional funding from the RJ Research Support Foundation enabled an expansion of the Nascer no Brasil II (NB II) survey sample to achieve statewide representativeness. This expansion was accomplished by increasing the number of pregnant women included in each selected hospital. Data analyses were conducted by the NB II central team in collaboration with the state-level research team. To the extent possible, the resulting articles examined differences in the care provided across the capital, the metropolitan region, and municipalities in the interior, in both the public and private sectors.

In its 125^th^ anniversary year, the Oswaldo Cruz Foundation continues to strengthen its national and international partnerships. RJ, where the Foundation is based, will serve as the institutional recipient of this analysis of obstetric care within the region. This territorial linkage facilitates the identification of both advances and persistent barriers in efforts to improve the health of pregnant women, mothers, and newborns, and may inform the development of targeted public strategies and policies.

This thematic issue includes seven articles. The first, "Prenatal Care Utilization and Hospital Structure According to Pregnant Women's Obstetric Risk," analyzes the adequacy of health service infrastructure in meeting the needs of pregnant women and those in labor; The second, "Sociogeographic Inequalities in Childbirth Care," highlights geographic and social disparities in the occurrence of vaginal births, linked to both facilitating and inhibiting factors; The third article, "Prenatal Care Adequacy According to Type of Childbirth Financing," discusses the contrast between high prenatal care coverage and its limited adequacy; The fourth article, which addresses obstetric violence, reveals a high prevalence of this issue in RJ, with marked sociodemographic inequities; The fifth, "Hospital Practices and Breastfeeding," explores the role of institutional support, care management, and the reduction of cesarean sections, among other factors, in promoting breastfeeding; The sixth article, "Postpartum Mental Disorders in the State of Rio de Janeiro," emphasizes the need for proper diagnosis of mental health conditions that often present with overlapping symptoms of depression, anxiety, and stress; and the final article, "Maternal and Perinatal Outcomes in Adolescents and Women with Advanced Maternal Age," shows that adolescents received lower-quality care compared to adult women, while those of advanced maternal age experienced a higher frequency of pregnancy-related pathologies.

An in-depth analysis of the determinants affecting the quality of labor and birth care in RJ aims to enhance understanding of the underlying causes of elevated maternal and perinatal mortality rates. This analysis sought to identify potential strategies for their reduction, thereby contributing to improved well-being for society and families across the city of Rio de Janeiro and the broader Fluminense region. Additionally, it may support Brazil in fulfilling its commitments to international organizations in achieving the Sustainable Development Goals by 2030.

Efforts to improve the health of the Brazilian population should prioritize the first two years of children's lives, as this period lays the foundation for their full physical, intellectual, and emotional development^12^.
