# Supplementary material for: Pregnancy, childbirth, and birth in the state of Rio de Janeiro
Source: Rev Saude Publica. 2025 Oct 20;59(Suppl 1):e2s. doi: 10.11606/s1518-8787.2025059supl1ap (PMC12539631; doi:10.11606/s1518-8787.2025059supl1ap)
Supplement: Translation

# Translation: Gestação, parto e nascimento no estado do Rio de Janeiro

**Authors:** Maria do Carmo Leal

**Affiliations:** I Fundação Oswaldo Cruz. Escola Nacional de Saúde Pública. Departamento de Métodos Quantitativos em Saúde. Rio de Janeiro, RJ, Brasil

O tema da Organização Mundial da Saúde para celebrar o Dia da Saúde, em 7 de abril, neste ano de 2025 foi "Começos saudáveis, futuros esperançosos", com o objetivo de fazer uma chamada aos governos e à comunidade da saúde sobre a prioridade para a redução das mortes maternas e neonatais evitáveis^1^. Mais que isso, o tema expande-se para abordar um conceito de saúde que extrapola a redução de óbitos e ancora-se na promoção da saúde da mulher e do seu bebê para assegurar um futuro promissor para ambos.

Temos verificado uma queda vertiginosa nas taxas de fecundidade das mulheres brasileiras, com diminuição do número de filhos tidos e aumento da escolaridade e do acesso ao mercado de trabalho^2,3^. Também estamos assistindo, nos últimos dez anos, a uma redução importante da gestação na adolescência e à postergação da maternidade, nas mulheres entre 20 e 49 anos^4^. Toda essa dinâmica da transição obstétrica em curso no país^5^ nos leva a imaginar que, com menor número de filhos e aumento da escolaridade e acesso ao trabalho, as mulheres brasileiras podem ter gestações mais saudáveis e gerar filhos também mais saudáveis.

Outro fator importante no resultado de uma gestação é o acesso adequado à atenção pré-natal e ao parto, com qualidade e respeito aos direitos sexuais e reprodutivos das mulheres. No Brasil o Sistema Único de Saúde, o SUS, oferta pelo menos uma consulta de pré-natal a 99% das mulheres, mas, considerando o critério do número adequado de consultas para a idade gestacional ao nascimento, esse percentual se reduz muito^6^, e assistência pré-natal insuficiente, ou ausente, se relaciona com muitos efeitos adversos para a puérpera e o recém-nascido^6^. Já a atenção ao parto, que atualmente ocorre majoritariamente em hospitais, tem cobertura de 98%, com alguma diferença regional para a Região Norte.

O cenário descrito sugere que, de modo geral, a maioria das mulheres está melhorando o padrão de vida e sendo atendida durante as suas gestações. Mas, ao analisar os coeficientes da mortalidade materna e perinatal do Brasil, verificamos que eles decresceram pouco nos últimos 20 anos^7^. Daí a importância de se fazer uma análise em profundidade sobre a gestação, parto e nascimento nos diversos contextos nacionais. Reconhecendo que as abissais iniquidades sociais do país têm papel importante nesses resultados, importa verificar se essas iniquidades também se refletem e se reproduzem na qualidade da atenção prestada nos serviços de saúde.

Políticas desenvolvidas pelo Ministério da Saúde para melhorar o atendimento ao parto em maternidades públicas, tais como o Programa de Humanização do Parto e Nascimento e a Rede Cegonha^8,9^, surtiram efeito na ampliação do acesso às tecnologias apropriadas ao parto, melhorando o conforto e ampliando o tratamento digno ofertado às gestantes e aos recém-nascidos^9^, mas estudos nacionais têm mostrado que o impacto dessas melhorias não se expressa na redução dos partos operatórios, que continuam numa curva ascendente, nem nas estatísticas de mortalidade^7,10^. Da mesma forma, iniciativas na saúde suplementar, como a criação do Programa Parto Adequado, têm apresentado resultados positivos, embora ainda incipientes^11^.

O número temático "Gestação, Parto e Nascimento no Estado do Rio de Janeiro" faz parte da Pesquisa Nacional sobre Aborto, Parto e Nascimento, conhecida como Nascer no Brasil II (NB II), financiada pelo Ministério da Saúde e pela Fundação Oswaldo Cruz, envolvendo 21.400 mulheres entrevistadas nas maternidades, com representação amostral das grandes regiões brasileiras (Norte, Nordeste, Sudeste, Sul e Centro-Oeste), tipo de pagamento do parto (público, privado e misto) e área geográfica (região metropolitana e interior).

No estado do Rio de Janeiro (ERJ), um financiamento da Fundação de Amparo à Pesquisa do ERJ permitiu expandir a amostra da pesquisa NB II, visando alcançar representatividade para o nível estadual. A ampliação da amostra ocorreu pelo aumento do número de gestantes participantes em cada hospital sorteado. As análises foram conduzidas pela equipe central da NB II, juntamente com a equipe de profissionais que realizaram a investigação em nível estadual. No limite da possibilidade, os artigos abordaram as diferenças na atenção ofertada no município da capital, na região metropolitana e nos demais municípios do interior, tanto pelo setor público como pelo privado.

A Fundação Oswaldo Cruz celebra neste ano os seus 125 anos, com estreitamento das parcerias nacionais e internacionais que a constitui. O ERJ, onde está situada, receberá essa análise da realidade da assistência obstétrica como parte dessa relação de pertencimento territorial, o que facilita a identificação dos avanços e das barreiras para a melhoria da saúde de gestantes, mães e recém-nascidos e pode orientar o desenvolvimento de estratégias e políticas públicas.

São sete artigos que compõem esse número temático. O primeiro deles, "Utilização do pré-natal e estrutura hospitalar segundo risco obstétrico da gestante", analisará a adequação da estrutura dos serviços de saúde às necessidades das gestantes e parturiente; o segundo, "Desigualdades sociogeográficas na atenção ao parto", mostra que há diferenças geográficas e sociais na ocorrência do parto vaginal associadas a fatores promotores e inibidores dessa prática; o terceiro, "Adequação do pré-natal segundo tipo de financiamento do parto", aborda a alta cobertura do pré-natal aliada à precária adequação dele; o quarto, sobre violência obstétrica, mostra a alta prevalência desse problema no ERJ, com iniquidades sociodemográficas; o quinto, "Práticas hospitalares e amamentação", aborda a importância do apoio, manejo e redução de cesarianas, entre outros fatores, para ampliação do aleitamento materno; o sexto, "Transtornos mentais no pós-parto no ERJ", chama a atenção para a necessidade de diagnóstico desse transtorno que pode cursar com quadros complexos, sobrepondo sintomas de depressão, ansiedade e estresse; e o último, "Desfechos maternos e perinatais em adolescentes e mulheres com idade materna avançada", mostra que as adolescentes receberam pior assistência em relação às adultas, enquanto as mulheres com idade materna avançada apresentaram maior frequência de patologias.

A análise dos fatores determinantes da qualidade da atenção ao parto e nascimento no ERJ em profundidade espera contribuir para o entendimento das causas das altas taxas de mortalidade materna e perinatal, indicar caminhos possíveis para sua redução e, dessa forma, contribuir para o aumento do bem-estar da sociedade e das famílias cariocas e fluminenses. Também pode colaborar para que o Brasil cumpra os seus compromissos assumidos com os organismos internacionais para o alcance dos Objetivos de Desenvolvimento Sustentável, em 2030.

Todos os esforços para a melhoria da saúde da população brasileira devem mirar nos dois primeiros anos de vida das crianças, pois aí são plantadas as sementes do desenvolvimento físico, intelectual e afetivo completo do ser humano^12^.
